# Detection and Classification of Breast Lesions With Readout-Segmented Diffusion-Weighted Imaging in a Large Chinese Cohort

**DOI:** 10.3389/fonc.2021.636471

**Published:** 2021-03-22

**Authors:** Zhen Lu Yang, Yi Qi Hu, Jia Huang, Chen Ao Zhan, Min Xiong Zhou, Xiao Yong Zhang, Hui Ting Zhang, Li Ming Xia, Tao Ai

**Affiliations:** ^1^Department of Radiology, Tongji Hospital, Tongji Medical College, Huazhong University of Science and Technology, Wuhan, China; ^2^College of Medical Imaging, Shanghai University of Medicine & Health Sciences, Shanghai, China; ^3^MR Scientific Marketing, Siemens Healthcare, Wuhan, China; ^4^MR Collaborations, Siemens Healthcare, Shenzhen, China

**Keywords:** breast neoplasms, magnetic resonance imaging, diffusion weighted MRI, sensitivity, specificity

## Abstract

**Objectives:** To evaluate the performance of readout-segmented echo-planar imaging DWI (rs-EPI DWI) in detecting and characterizing breast cancers in a large Chinese cohort with comparison to dynamic contrast-enhanced MRI (DCE-MRI).

**Methods:** The institutional review board approved this retrospective study with waived written informed consent. A total of 520 women (mean age, 43.1- ± 10.5-years) were included from July 2013 to October 2019. First, the ability of rs-EPI DWI in detecting breast lesions identified by DCE-MRI was evaluated. The lesion conspicuity of rs-EPI-DWI and DCE-MRI was compared using the Wilcoxon signed rank test. With pathology as a reference, the performance of rs-EPI DWI and DCE-MRI in distinguishing breast cancers was evaluated and compared using the Chi-square test.

**Results:** Of 520 women, 327/520 (62.9%) patients had 423 lesions confirmed by pathology with 203 benign and 220 malignant lesions. The rs-EPI DWI can detect 90.8% (659/726) (reader 1) and 90.6% (663/732) (reader 2) of lesions identified by DCE-MRI. The lesion visibility was superior for DCE-MRI than rs-EPI-DWI (all *p* < 0.05). With pathology as a reference, the sensitivities and specificities of rs-EPI DWI in diagnosing breast cancers were 95.9% (211/220) and 85.7% (174/203) for reader 1 and 97.7% (215/220) and 86.2% (175/203) for reader 2. No significant differences were found for the performance of DCE-MRI and rs-EPI DWI in discriminating breast cancers (all *p* > 0.05).

**Conclusions:** Although with an inferior lesion visibility, rs-EPI DWI can detect about 90% of breast lesions identified by DCE-MRI and has comparable diagnostic capacity to that of DCE-MRI in identifying breast cancer.

## Key Points

- Readout-segmented echo-planar imaging DWI (rs-EPI DWI) can detect about 90% of breast lesions identified by dynamic contrast-enhanced MRI (DCE-MRI).
- With pathology as reference, the sensitivity and specificity of rs-EPI DWI in characterizing breast cancers were 95.9% (211/220) and 85.7% (174/203) for reader 1 and 97.7% (215/220) and 86.2% (175/203) for reader 2.
- No significant differences were found between rs-EPI DWI and DCE-MRI for the sensitivity, specificity, accuracy, positive predictive value, and negative predictive value in distinguishing breast cancers (all *p* > 0.05).

## Introduction

Breast cancer is the most common cancer for women worldwide and has become the leading cause of cancer-related death in Chinese women younger than 45-years old (1, 2). Chinese patients contribute significantly to the global burden of breast cancer and related deaths given the large population (1, 3). Miller et al. (4) reported that the 5-year relative survival rates for patients with breast cancer at stage I and stage IV were 100 and 26%, respectively. Early detection and treatment are crucial for improving the prognosis of patients with breast cancer.

Currently, mammography is recommended by clinical guidelines for breast cancer screening in many Western countries for women older than 40-years (5–7). However, Asian women usually have relatively dense and small breasts, making it difficult to effectively detect lesions in these women with mammography alone (7). Dynamic contrast-enhanced MRI (DCE-MRI) is so far the most sensitive imaging modality for identifying breast cancers, and it is therefore recommended for cancer screening of high-risk women as a supplement to mammography and/or breast ultrasound (8, 9). However, several disadvantages prevent its widespread use in screening average-risk women, including intravenous injection of gadolinium-based contrast agents (GBCAs), higher cost, longer acquisition time, and lower availability (10, 11). Abbreviated breast MRI protocols have been proposed to overcome some of these limitations and show feasibility in MRI breast cancer screening (12, 13). However, the gadolinium deposition in the body due to repeated injection of GBCAs has attracted broad attention over the world (14), which makes DCE-MRI unreasonable for breast cancer screening in the general population.

In order to identify a safe and effective screening tool, many studies have considered using non-contrast MRI protocols based on diffusion-weighted imaging (DWI) (10, 11, 15–17). In early studies, conventional single-shot echo-planar imaging DWI (ss-EPI DWI) sequences were used offering an advantage of speed and no requirement for GBCA contrast. However, it suffered from susceptibility artifacts, geometric distortions, and spatial blurring (18–21), which partly contributed to the discrepant and unsatisfactory sensitivities and specificities of DWI for breast cancer detection (17, 22, 23). Pinker et al. concluded that conventional ss-EPI DWI was not sufficient as a stand-alone modality for breast cancer detection (11). DWI based on readout-segmented technique (a multi-shot strategy) may improve spatial resolution for superior sensitivity and/or specificity and provide more potential when combined with a new technique (24, 25). During diffusion encoding in readout-segmented echo-planar imaging (rs-EPI), each shot involves only a limited transversal of k-space in the readout direction, but full resolution along the phase encoding direction (26). rs-EPI DWI should improve the visualization of anatomic structures with less image distortion and superior spatial resolution (19, 27, 28). Recently, the consensus recommendations of the European Society of Breast Radiology (EUSOBI) breast DWI working group stated that breast DWI had high specificity and may improve lesion classification in cancer screening. However, evidence supporting the use of DWI for screening as a stand-alone test or as a part of an unenhanced MRI protocol is currently insufficient (29).

The purpose of this study was to evaluate the ability of rs-EPI DWI in detecting breast lesions identified by DCE-MRI and the performance of rs-EPI DWI in distinguishing breast cancers with comparison to DCE-MRI in a large Chinese cohort by using pathology as the reference standard.

## Materials and Methods

### Patients

The institutional review board of our hospital approved this single-institution retrospective study. The written informed consents of patients were waived. From July 2013 to October 2019, 956 women (mean age, 43.2- ± 10.5-years) were referred for breast MRI in our hospital due to one of the following conditions (inclusion criteria): (a) suspicious lesions on mammography and/or ultrasonography; (b) clinical symptoms/signs, such as breast pain, mass, and abnormal changes of skin and nipple; (c) high risk of breast cancer; and (d) presurgical evaluation or baseline assessment for monitoring therapeutic response.

The exclusion criteria included: (a) previous treatments including surgery, radiotherapy, and chemotherapy (patients underwent MRI for the assessment of therapy response or recurrence, *n* = 247); (b) needle biopsy performed prior to the breast MRI (*n* = 148); (c) patients with breast implants (*n* = 28); (d) poor image quality due to marked motion artifacts and/or insufficient field of view (*n* = 3); (e) only nipple lesions without involving breast parenchyma (*n* = 3); (f) pregnancy or lactation (*n* = 7); and (g) simple cysts (as a per-lesion exclusion). Finally, a total of 520 women (mean age, 43.1- ± 10.5-years) were included in this study.

**Figure 1:**
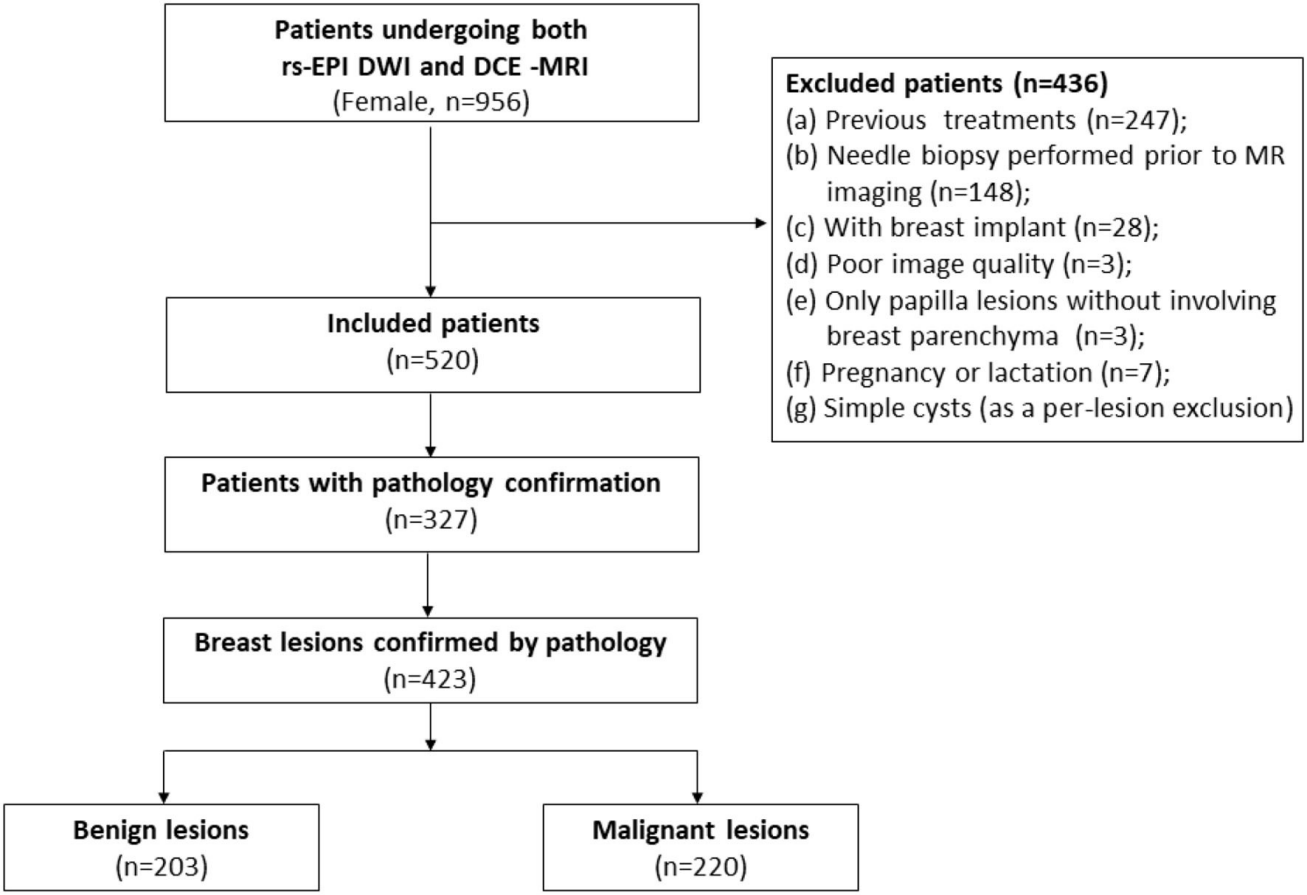
Flowchart of this study. DCE-MRI, dynamic contrast material-enhanced MRI; rs-EPI DWI, readout-segmented echo-planar imaging diffusion-weighted imaging.

Medical records were reviewed to record corresponding pathology results and status of estrogen receptor (ER), progesterone receptor (PR), human epidermal growth factor receptor-2 (HER2), and Ki-67 if available. The flowchart of this study is depicted in Figure 1.

### Imaging Protocols

All breast MR images were obtained using a 3T MRI scanner (MAGNETOM Skyra, Siemens Healthcare, Erlangen, Germany) with bilateral, dedicated 4- or 16-channel phased-array breast coil with patients in the prone position. The scanning protocol mainly included T2-weighted imaging, rs-EPI DWI, and DCE-MRI. For DWI scanning in this study, 4 b values (0, 50, 1,000, and 2,000 s/mm^2^) were used. The imaging parameters of each sequence are described in Table 1. For all DCE-MRI protocols, the gadodiamide contrast medium (Omniscan, GE Healthcare, Milwaukee, WI, USA) was intravenously injected at the end of the third dynamic acquisition phase, with a dose of 0.1 mmol/kg body weight at 2.5 ml/s. Contrast administration was followed with a 20 ml saline flush.

**Table 1:** Sequence parameters for T2-weighted imaging, diffusion-weighted imaging, and dynamic contrast-enhanced MRI.

			**Dynamic contrast-enhanced MRI**		
**Parameters**	**T2-weighted sequence**	**Readout-segmented echo-planar imaging diffusion-weighted sequence (RESOLVE)**	**TWIST-VIBE with** **35 phases** **(*n* = 61, from Jul. 2013 to Jun. 2015)**	**TWIST-VIBE with** **28 phases** **(*n* = 184, from Jul. 2015 to Oct. 2017)**	**TWIST-VIBE with** **60 phases** **(*n* = 275, from Nov. 2017 to Oct. 2019)**
Repetition time (ms)	3,700	5,000	5.40	5.91	5.24
Echo time (ms)	101	70	2.46	2.46	2.46
Field of view (mm^2^)	320 × 320	169 × 280	270 × 320	290 × 320	260 × 320
Matrix	224 × 320	114 × 188	243 × 320	203 × 320	182 × 320
Flip angle (°)	137	180	9	10	10
Slice thickness (mm)	4.0	5.0	1.5, no gap	1.5, no gap	1.5, no gap
Pixel bandwidth (Hz/Px)	347	887	980	780	780
Parallel imaging	GRAPPA (x2)	GRAPPA (x2)	CAIPIRINHA (x4)	CAIPIRINHA (x4)	CAIPIRINHA (x4)
b-values (sec/mm^2^)		0, 50, 1,000, 2,000			
Diffusion acquisition		5 readout segments, 1 average			
Diffusion gradient mode		3-scan-trace			
Temporal resolution (sec/phase)			11.24	7.96 (12 s of time interval for the late 10 phases)	5.74
Acquisition time (min:s)	2:06	4:27	6:48	5:51	5:57

*TWIST, time-resolved angiography with stochastic trajectories; VIBE, volume-interpolated breath-hold examination*.

### Image Assessment

All image datasets were reviewed using software RadiAnt DICOM-Viewer (version 5.0.2, Medixant, Poznán, Poland) by two readers (TA and ZLY with 10 and 3-years of experience in the breast MRI interpretation, respectively). Each reviewer was blinded to the corresponding clinical information, other imaging results, and pathology reports.

For DCE-MRI, the two readers independently evaluated images and determined the lesion types (mass or non-mass), lesion locations (by clock position), the distance of the lesions from the nipple, and maximal trans-axial diameters (only for mass lesions). For multiple lesions of the ipsilateral breast, a “separate” lesion was identified if the lesion location was relatively separate, and its boundary was disconnected/not continuous with other lesions. The amount of fibroglandular tissue (FGT) and background parenchymal enhancement (BPE) was also recorded by two readers by consensus according to the fifth edition of the Breast Imaging Reporting and Data System (BI-RADS® 5th edition) (30). The mean signal intensity of a region of interest (ROI) in each phase from 35/28/60 phases (all phases were involved) was used to generate a time-signal intensity curve (TIC) for each lesion by using a dedicated Syngo MR Workstation (Siemens Healthcare, Erlangen, Germany) with software program “Mean Curve.” (Siemens Healthcare, Erlangen, Germany) An ROI for each lesion was manually drawn with an area of 0.2–0.4 cm^2^ by avoiding vessels and necrotic regions. The BI-RADS categories of lesions on DCE-MRI were performed by referring criteria described in Supplementary Table 1, and reasonable adjustment was allowed according to the experience of readers. In brief, lesions were categorized as BI-RADS 2 or 5 when meeting all benign suspicious or malignant suspicious criteria, respectively. In case of fulfilling only one or more than one malignant suspicious criteria, BI-RADS 3 or 4 were given, respectively. The lesions with BI-RADS 2 or 3 were regarded as benign lesions; and the lesions with BI-RADS 4 or 5 were regarded as malignant lesions.

For rs-EPI DWI, the two readers independently analyzed the DWI images with different b-values and apparent diffusion coefficient (ADC) maps to record the lesion types (mass or non-mass), lesion locations (by clock position), the distance of the lesions from the nipple, and BI-RADS categories. The criteria of identifying lesion type on rs-EPI DWI was similar to that on DCE-MRI according to BI-RADS® 5th edition (30). T2-weighted MR images were included in DWI-based evaluation to exclude simple cysts. Mean ADC values were calculated using an in-house developed software called body diffusion laboratory on basis of a computing language and interactive environment (BoDiLab, Siemens Healthineers, Erlangen, Germany) as described in prior studies (31). All *b*-value data (0, 50, 1,000, and 2,000 s/mm^2^) were used for generating ADC maps by using the following equation: S(*b*) = S0 × exp (–*b* × ADC), where S(*b*) is the DWI signal intensity at a certain *b*-value, S(0) is the baseline signal at *b* = 0, and *b* is the applied diffusion sensitization. For these measurements, an ROI for each lesion (0.2–0.4 cm^2^) was drawn manually on the darkest portion of the ADC map by avoiding fatty and necrotic tissues by referring to corresponding T2-weighted images (29). The previously reported ADC cutoff values of 1.25 × 10^−3^ mm^2^/s, which produced an excellent diagnostic accuracy (16), were used to distinguish malignant from benign lesions. The BI-RADS categories of lesions on rs-EPI DWI were referred to the criteria in Supplementary Table 1 with the same rules mentioned in DCE-MRI assessment's subsection.

Reader 2 (ZLY) was responsible for matching lesions on DCE-MRI and rs-EPI DWI according to lesion size, location, and distance of the lesion from the nipple. The lesions on rs-EPI DWI or DCE-MRI were also correlated with the corresponding pathological findings according to the lesion locations described in the surgery/needle biopsy records and detailed pathology reports.

Additionally, the lesion visualization (lesion conspicuity) on DCE-MRI and on rs-EPI DWI with *b*-value of 1,000 s/mm^2^ was evaluated independently by two readers using a 3-point scale: 3-excellent (clearly showing lesions and its detailed morphological features); 2-good (clearly showing lesions, but with loss of anatomic details); and 1-poor (barely showing lesions with unsatisfactory contrast).

### Statistical Analysis

Statistical Package for the Social Sciences (SPSS) version 21.0 (IBM, Armonk, NY, USA) was applied for statistical analysis. The continuous variable was shown as mean ± SD, and categorical variable was displayed as percentage.

For summarizing lesion characteristics between benign and malignant lesions, data recorded by reader 2 (TA, who was more experienced in interpreting the breast MRI) was used for analysis, including lesion size, shape, margin, internal enhancement, distribution of non-mass-like lesions, TIC, and mean ADC value. Those characteristics were compared using the Student's *t*-test or the Chi-square test between benign and malignant lesions groups.

The ability of rs-EPI DWI in detecting breast lesions identified by DCE-MRI was evaluated on a per-patient and per-lesion level, respectively. Then, with pathology results as a reference, the performance of rs-EPI DWI and DCE MRI in distinguishing breast lesions was assessed on per-patient and per-lesion basis and was compared by using the Chi-square test. The inter-reader agreement for lesion visualization on rs-EPI DWI (readers 1 and 2) and DCE-MRI (readers 1 and 2) was, respectively, assessed by the Cohen's Kappa analysis: κ = 0.81–1.00, excellent agreement; κ = 0.61–0.80, good agreement; κ = 0.41–0.60, moderate agreement; κ = 0.21–0.40, fair agreement; κ = 0.01–0.20, slight agreement; and κ = 0, no agreement (32). Additionally, the lesion conspicuity between rs-EPI-DWI and DCE-MRI was compared using the Wilcoxon signed rank test. When a *p* < 0.05, a statistical significance was considered. Based on available data, mean ADC values of invasive breast cancers with different molecular subtypes were compared by the one-way ANOVA test or by the Student *t*-test. The molecular subtypes of breast cancers include luminal A (ER or PR positive, or both, HER2 negative, and low expression of Ki-67), luminal B (ER or PR positive, or both, HER2 negative, and high expression of Ki-67), HER2-enriched (HER2 positive), and triple-negative tumors (ER, PR, and HER2 negative) (33).

## Results

### General Characteristics

Of 520 women (mean age, 43.1- ± 10.5-years), FGT was observed in 21.7% (113/520) patients with low density (a and b) and 78.3% (407/520) patients with high density (c and d). Of patients with high density, 58.2% (237/407) were older than 40-years. Minimal or mild BPE was observed in 61.9% of patients (322/520), and moderate or marked BPE was observed in 38.1% of patients (198/520) (Table 2). Of 520 patients, 327/520 (62.9%) patients had 423 breast lesions confirmed by pathology with 203 benign lesions and 220 malignant lesions (Table 2). The lesion characteristics of benign and malignant lesions are shown in Supplementary Table 2.

**Table 2:** Characteristics of 520 women study cohort.

**Characteristic**	**Result**
**Mean age (years)**	43.1 ± 10.5, Range of 12–83
**Amount of FGT**	
Almost entirely fat (a)	14 (2.7%)
Scattered fibroglandular tissue (b)	99 (19.0%)
Heterogeneous fibroglandular tissue (c)	324 (62.3%)
Extreme fibroglandular tissue (d)	83 (16.0%)
**BPE level**	
Minimal	60 (11.5%)
Mild	262 (50.4%)
Moderate	164 (31.5%)
Marked	34 (6.5%)
**Available pathology results**	
**Patients**	327
Benign	120/327 (36.7%)
Malignant	207/327 (63.3%)
**Breast lesions**	423
Benign	203/423 (48.0%)
Mass-like	192/423 (45.4%)
Non-mass-like	11/423 (2.6%)
Malignant	220/423 (52.0%)
Mass-like	181/423 (42.8%)
Non-mass-like	39/423 (9.2%)

Data percentages in parentheses. Mean age is mean ± SD.

*DCE-MRI, dynamic contrast-enhanced MRI; FGT, fibroglandular tissue; BPE, background parenchymal enhancement*.

### Detection Ability of rs-EPI DWI for Breast Lesions Identified by DCE-MRI

**Figure 2:**
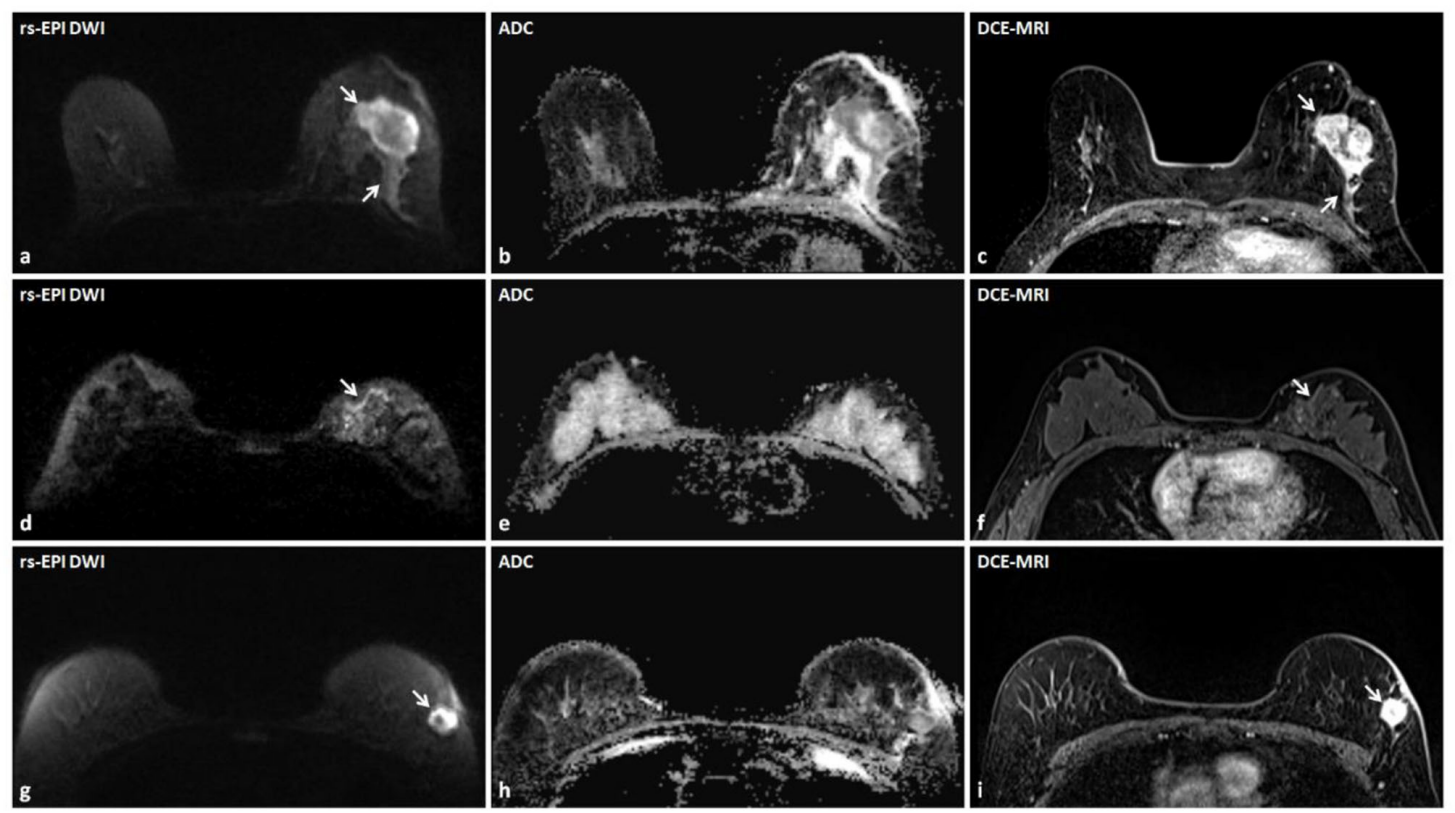
Three lesions accurately detected by rs-EPI DWI with detailed morphology characteristics in three patients. **(a–c)** rs-EPI DWI (*b*-value, 1,000 s/mm^2^), ADC map, and DCE-MRI of a 60-year-old woman with the left breast invasive carcinoma. **(a)** rs-EPI DWI shows an irregular mass (arrow) with markedly low signal on ADC map **(b)**, and the lesion shape and extent are consistent with that delineated on DCE-MRI (arrow) **(c)**. **(d–f)** rs-EPI DWI (*b*-value, 1,000 s/mm^2^), ADC map, and DCE-MRI of a 50-year-old woman with the left breast ductal carcinoma *in situ*. **(d)** rs-EPI DWI shows abnormal linear hyper-intensity distributed along the duct (arrow) with superior visualization than observed on DCE-MRI (arrow) **(f)**, which may reflect the distribution of ductal carcinoma *in situ*. **(g–i)** rs-EPI DWI (*b*-value, 1,000 s/mm^2^), ADC map, and DCE-MRI of a 57-year-old woman with the left breast invasive carcinoma. **(g)** rs-EPI DWI shows a mass with the heterogeneous internal structure (arrow) and low signal in the rim on ADC map **(h)**. **(i)** DCE-MRI shows an irregular mass (arrow) with heterogeneous enhancement.

On DCE-MRI, reader 1 detected 726 breast lesions (<10 mm, *n* = 293; ≥10 mm, *n* = 368; non-mass-like, *n* = 65) in 433 patients (low breast density, *n* = 93; high breast density, *n* = 340). The reader 2 diagnosed 732 breast lesions (<10 mm, *n* = 299; ≥10 mm, *n* = 368; non-mass-like lesions, *n* = 65) in 437 patients (low breast density, *n* = 93; high breast density, *n* = 344). The rs-EPI DWI can detect 95.4% (413/433) of patients and 90.8% (659/726) of lesions identified by DCE-MRI by reader 1, and 95.4% (417/437) of patients and 90.6% (663/732) of lesions depicted by DCE-MRI by reader 2. Of lesions ≥10 mm on DCE-MRI, 96.2% (354/368) and 96.2% (354/368) can be detected on rs-EPI DWI by reader 1 and reader 2, respectively. For lesions <10 mm on DCE-MRI, rs-EPI DWI can depict 82.6% (242/293) and 82.3% (246/299) of lesions by reader 1 and reader 2, respectively. Figure 2 shows lesions delineated by rs-EPI DWI with good visualization of morphological details.

A good or excellent lesion visualization (2 or 3 score) was given in 94.0% (640/681) of lesions by reader 1 and 92.7% (636/686) by reader 2 on rs-EPI DWI, and 97.4% (707/726) of lesions by reader 1 and 97.0% (710/732) by reader 2 on DCE-MRI. The inter-reader agreement of the lesion visualization evaluation was good on rs-EPI-DWI (*k* = 0.780) and on DCE-MRI (*k* = 0.683). The lesion visibility was superior for DCE-MRI than rs-EPI-DWI (all *p* < 0.05).

### Discrepant Findings of DCE-MRI and rs-EPI DWI in Detecting Breast Lesions

**Figure 3:**
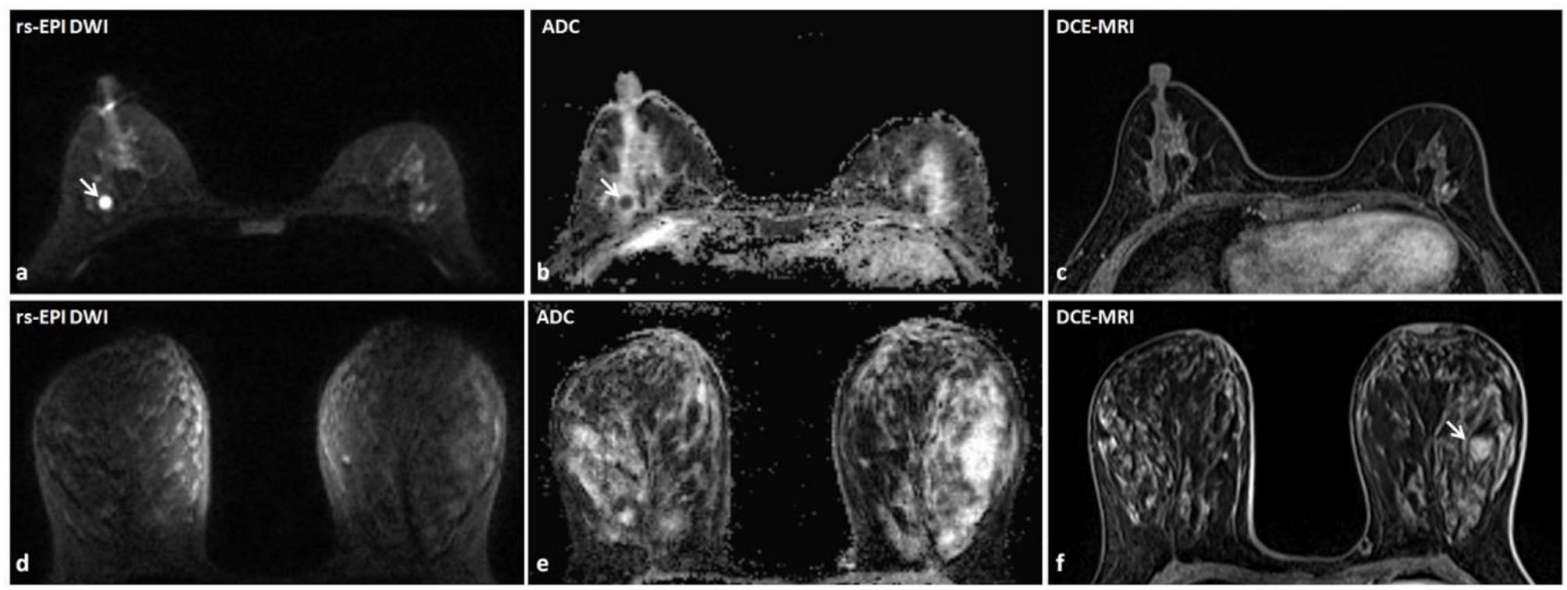
Discrepant findings of DCE-MRI and rs-EPI DWI for detecting breast lesions. **(a)** rs-EPI DWI (*b*-value, 1,000 s/mm^2^) shows a round, well-defined, and homogeneous nodule (6.7 mm) (arrow) with markedly low signal on ADC map **(b)** (mean ADC value, 0.47 × 10^−3^ mm^2^/s) (arrow) in the right breast of a 43-year-old woman, whereas **(c)** DCE-MRI shows no abnormal enhancement at that location. This lesion was pathologically verified as the right breast fibroadenosis. **(d–f)** rs-EPI DWI (*b*-value, 1,000 s/mm^2^), ADC map, and DCE-MRI of a 47-year-old woman with the left breast adenosis. **(f)** DCE-MRI depicts a well-defined lesion (14.6 mm) (arrow), whereas there is no abnormal signal on rs-EPI DWI **(d)** and ADC map **(e)**.

The details of discrepant findings of DCE-MRI and rs-EPI DWI in detecting breast lesions by two readers are shown in Table 3. A total of 22 lesions in 16 patients (reader 1) and 23 lesions in 17 patients (reader 2) were positive detection on rs-EPI DWI, whereas negative on DCE-MRI. A majority of those lesions were rated as BI-RADS 2 or 3 on rs-EPI DWI by the two readers and without any malignant pathology reports (Table 3, Figures 3a–c).

**Table 3:** Discrepant findings of DCE-MRI and rs-EPI DWI in detecting breast lesions.

**Findings**	**Age (y)**	**Size (mm)**	**Mass**	**Non-mass**	**BI-RADS ratings**		**BI-RADS ratings**		**Histopathological**		
	**(Mean ± SD)**	**(Mean ± SD)**	**(*n*)**	**(*n*)**	**(DCE-MRI)**		**(rs-EPI DWI)**		**results**		
					**2 or 3** **(*n*)**	**4 or 5** **(*n*)**	**2 or 3** **(*n*)**	**4 or 5** **(*n*)**	**Malignant** **(*n*)**	**Benign** **(*n*)**	**NA** **(*n*)**
**DCE-MRI (-) and rs-EPI DWI (+)**											
R1 (*n* = 22)	43.6 ± 6.8	6.9 ± 2.5	21	1			19	3	0	5	17
R2 (*n* = 23)	43.8 ± 6.6	6.8 ± 2.4	22	1			18	5	0	5	18
**DCE-MRI (+) and rs-EPI DWI (–)**											
R1 (*n* = 67)	42.1 ± 10.3	8.0 ± 4.6	65	2	62	5			2	23	42
R2 (*n* = 69)	42.1 ± 10.3	7.9 ± 4.5	67	2	64	5			2	23	44

*SD, standard deviation; BI-RADS, Breast Imaging Reporting and Data System; rs-EPI DWI, readout-segmented echo-planar imaging diffusion-weighted imaging; DCE-MRI, dynamic contrast-enhanced MRI; NA, not applicable; R1, reader 1; R2, reader 2*.

**Figure 4:**
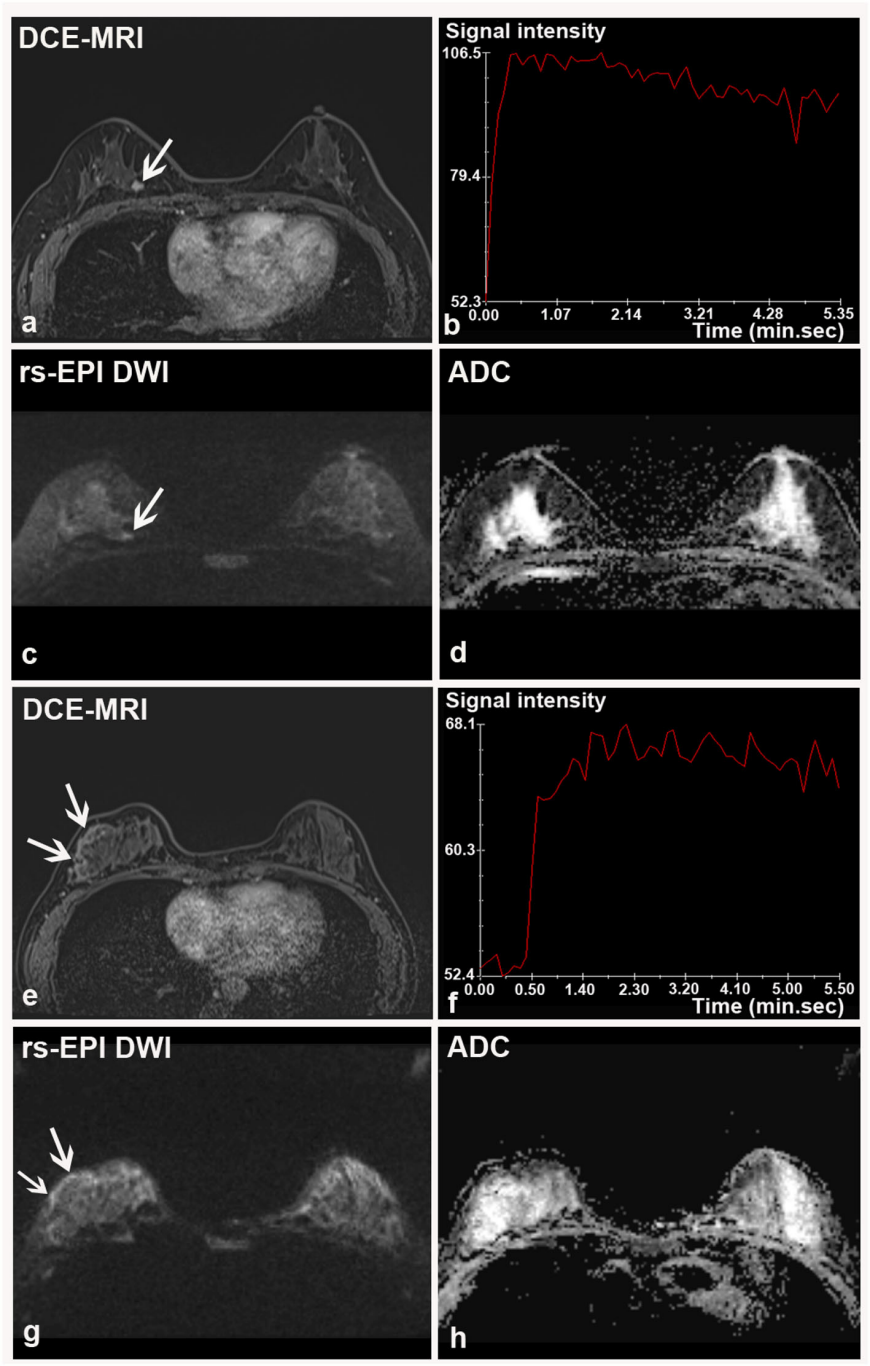
Two breast malignancies missed by rs-EPI DWI in two patients. **(a–d)** DCE-MRI, time-signal intensity curve (TIC), rs-EPI DWI (*b*-value, 1,000 s/mm^2^), and ADC map of a 55-year-old woman with the right breast ductal carcinoma *in situ*. **(a)** DCE-MRI shows a lobulated and spiculated nodule (8.7 mm) (arrow) with initial fast enhancement followed by a washout **(b)** classified as BI-RADS 4. No lesion was found on corresponding rs-EPI DWI **(c)** and ADC map **(d)**. **(e–h)** DCE-MRI, TIC, rs-EPI DWI (*b*-value, 1,000 s/mm^2^), and ADC map of a 42-year-old woman with the right breast ductal carcinoma *in situ*. **(e)** DCE-MRI shows non-mass-like enhancement along the parenchyma surface (arrow) with initial fast enhancement followed by plateau **(f)** classified as BI-RADS 4. No lesion can be identified on corresponding rs-EPI DWI **(g)** and ADC map **(h)**. Slight high signal can be retrospectively observed for both cases on rs-EPI DWI (**c,g**, respectively) (arrow), but it was not considered sufficient to confirm the presence of lesions. The latter finding may be attributable to the inferior spatial resolution (5 mm) of our rs-EPI DWI protocol relative to DCE-MRI protocol.

A total of 67 lesions in 56 patients (reader 1) and 69 lesions in 57 patients (reader 2) were positive on DCE-MRI, whereas negative on rs-EPI DWI (Table 3). Among those lesions, 76.1% (51/67) (reader 1) and 76.8% (53/69) (reader 2) had maximal diameter smaller than 10 mm, and more than 90% were categorized as BI-RADS 2 or 3 on DCE-MRI. According to the available pathological results, 34.3% (23/67) (reader 1) and 33.3% (23/69) (reader 2) of lesions missed by rs-EPI DWI were benign diseases (Figures 3d–f), and only two lesions were confirmed as malignant (Figure 4).

### Performance of rs-EPI DWI and DCE-MRI for Diagnosing Breast Cancers

With pathology as a standard reference, the performances of DCE-MRI and rs-EPI DWI for identifying breast cancers on per-patient basis and per-lesion basis are shown in Supplementary Table 3 and Table 4, respectively.

**Table 4:** Diagnostic performance of rs-EPI DWI and DCE-MRI for characterizing the breast cancers with pathology as reference standard.

	**Results (*****n*****)**				**Test performance (%)**				
**Imaging modality**	**TP**	**TN**	**FP**	**FN**	**Sens**.	**Spec**.	**PPV**	**NPV**	**Acc**.
**rs-EPI DWI**									
**Overall**									
R1	211	174	29	9	95.9 (211/220) [92.4–97.8]	85.7 (174/203) [80.2–89.9]	87.9 (211/240) [83.2–91.5]	95.1 (174/183) [90.9–97.4]	91.0 (385/423) [87.9–93.4]
R2	215	175	28	5	97.7 (215/220) [94.8–99.0]	86.2 (175/203) [80.8–90.3]	88.5 (215/243) [83.9–91.9]	97.2 (175/180) [93.7–98.8]	92.2 (390/423) [89.3–94.5]
**Mass-like lesion**									
R1	174	170	22	7	96.1 (174/181) [92.2–98.1]	88.5 (170/192) [83.3–92.3]	88.8 (174/196) [83.6–92.5]	96.0 (170/177) [92.1–98.1]	92.2 (344/373) [89.1–94.5]
R2	177	172	20	4	97.8 (177/181) [94.5–99.1]	89.6 (172/192) [84.5–93.2]	89.8 (177/197) [84.8–93.3]	97.7 (172/176) [94.3–99.1]	93.6 (349/373) [90.6–95.6]
**Non-mass-like lesion**									
R1	37	4	7	2	94.9 (37/39) [83.1–98.6]	36.4 (4/11) [15.2–64.6]	84.1 (37/44) [70.6–92.1]	66.7 (4/6) [30.0–90.3]	82.0 (41/50) [69.2–90.2]
R2	38	3	8	1	97.4 (38/39) [86.8–99.6]	27.3 (3/11) [9.7–56.6]	82.6 (38/46) [69.3–90.9]	75.0 (3/4) [30.1–95.4]	82.0 (41/50) [69.2–90.2]
**DCE-MRI**									
**Overall**									
R1	216	172	31	4	98.2 (216/220) [95.4–99.3]	84.7 (172/203) [79.1–89.0]	87.4 (216/247) [82.7–91.0]	97.7 (172/176) [94.3–99.1]	91.7 (388/423) [88.7–94.0]
R2	218	164	39	2	99.1 (218/220) [96.8–99.8]	80.8 (164/203) [74.8–85.6]	84.8 (218/257) [79.9–88.7]	98.8 (164/166) [95.7–99.7]	90.3 (382/423) [87.1–92.8]
**Mass-like lesion**									
R1	177	167	25	4	97.8 (177/181) [94.5–99.1]	87.0 (167/192) [81.5–91.0]	87.6 (177/202) [82.4–91.5]	97.7 (167/171) [94.1–99.1]	92.2 (344/373) [89.1–94.5]
R2	179	161	31	2	98.9 (179/181) [96.1–99.7]	83.9 (161/192) [78.0–88.4]	85.2 (179/210) [79.8–89.4]	98.8 (161/163) [95.6–99.7]	91.2 (340/373) [87.8–93.6]
**Non-mass-like lesion**									
R1	39	5	6	0	100 (39/39) [91.0–100]	45.5 (5/11) [21.3–72.0]	86.7 (39/45) [73.8–93.7]	100 (5/5) [56.6–100]	88.0 (44/50) [76.2–94.4]
R2	39	3	8	0	100 (39/39) [91.0–100]	27.3 (3/11) [9.7–56.6]	83.0 (39/47) [69.9–91.1]	100 (3/3) [43.9–100]	84.0 (42/50) [71.5–91.7]

TP, true positive; TN, true negative; FP, false positive; FN, false negative; Sens., sensitivity; Spec., specificity; PPV, positive predictive value; NPV, negative predictive value; Acc., Accuracy; R1, reader 1; R2, reader 2; rs-EPI DWI, readout-segmented echo-planar imaging diffusion-weighted imaging; DCE-MRI, dynamic contrast-enhanced MRI.

*Data in parentheses are the numerator and denominator. Data in brackets are 95% CIs*.

The sensitivity, specificity, and accuracy of rs-EPI DWI in distinguishing breast cancers on per-lesion level were 95.9% (211/220), 85.7% (174/203), and 91.0% (385/423) for reader 1, and 97.7% (215/220), 86.2% (175/203), and 92.2% (390/423) for reader 2. The sensitivity, specificity, and accuracy of DCE-MRI in diagnosing breast cancers on per-lesion level were 98.2% (216/220), 84.7% (172/203), and 91.7% (388/423) for reader 1 and 99.1% (218/220), 80.8% (164/203), and 90.3% (382/423) for reader 2. There were no significant differences for the overall performance in distinguishing breast cancers from benign lesions between DCE-MRI and rs-EPI DWI, and also for the analysis of the subgroups with different lesion types (all *p* > 0.05).

Based on the available data, the mean ADC values of the invasive breast cancers with different molecular subtypes are shown in Supplementary Table 4. A higher ADC value was found for non-luminal tumors when compared with luminal tumors.

### False Findings Depicted by rs-EPI DWI During Diagnosing Breast Cancers

**Figure 5:**
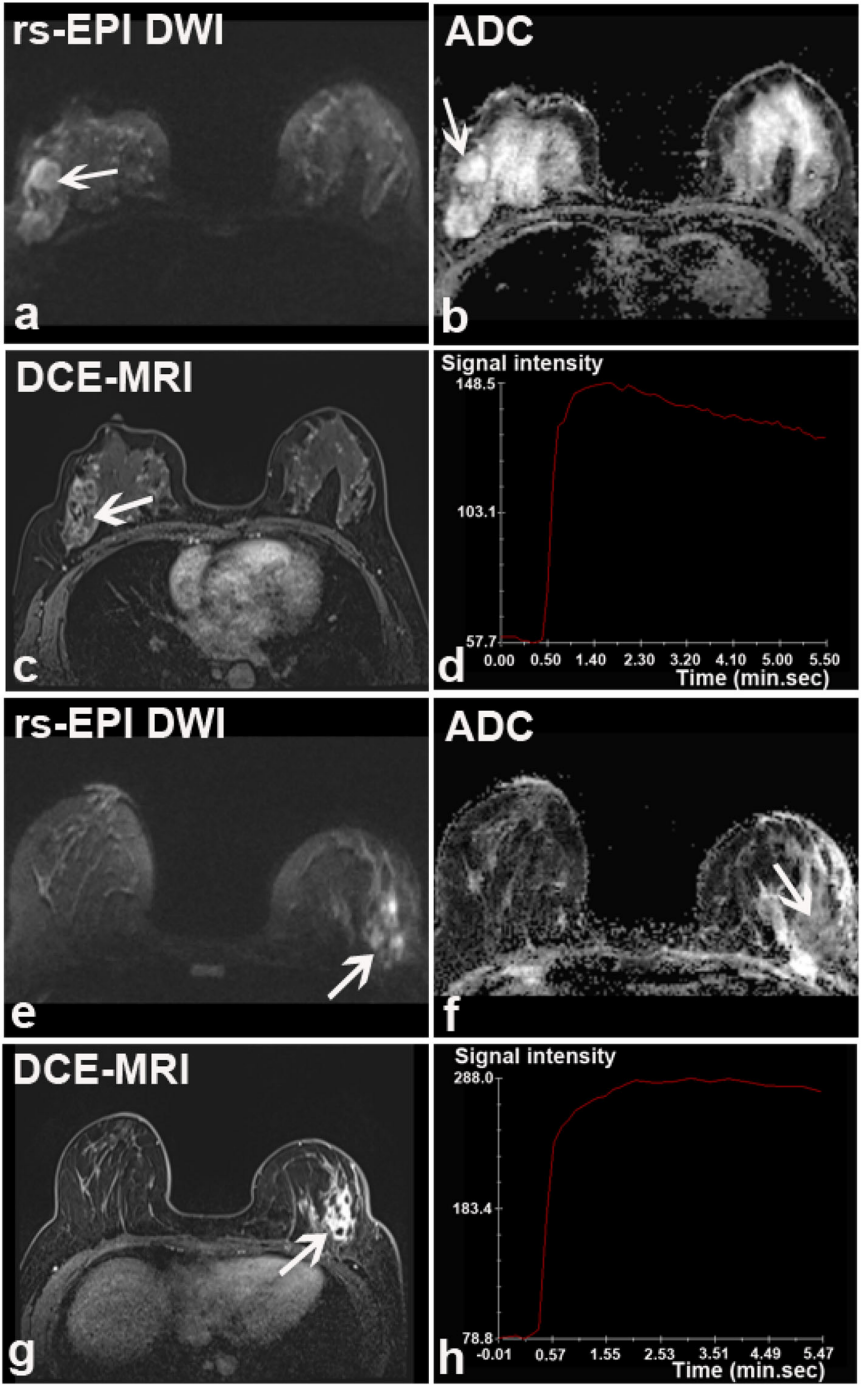
Two breast lesions falsely classified by rs-EPI DWI in two patients. **(a–d)** rs-EPI DWI (*b*-value, 1,000 s/mm^2^), ADC map, DCE-MRI, and time-signal intensity curve (TIC) from a 46-year-old woman with the right breast mucinous carcinoma. **(a)** rs-EPI DWI shows a lesion with an irregular shape and heterogeneous internal structure, but high signal on ADC map (arrow) **(b)**. The lesion was considered as fibrocystic hyperplasia and rated as BI-RADS 3. **(c)** DCE-MRI shows that this lesion has an irregular shape and heterogeneous signal enhancement (arrow) with initial fast enhancement followed by washout **(d)**. Thus, lesion was categorized as BI-RADS 4. **(e–h)** rs-EPI DWI (*b*-value, 1,000 s/mm^2^), ADC map, DCE-MRI, and TIC from a 33-year-old woman with the left breast granulomatous mastitis accompanying a small abscess. **(e)** rs-EPI DWI shows irregular high signals with markedly low signal on ADC map (arrow) **(f)**. Lesion was categorized as malignancy based upon rs-EPI DWI. **(g)** DCE-MRI shows non-mass-like enhancement with segmental distribution (arrow) and initial fast enhancement followed by plateau **(h)**, thus categorized as BI-RADS 4.

Several malignant tumors were classified as benign diseases based upon rs-EPI DWI including invasive carcinoma (*n* = 5 and 3 for readers 1 and 2, respectively), ductal carcinoma *in situ* (*n* = 3 and 2), and mucinous carcinoma (*n* = 1 and 0) (Figures 5a–d). A total of 29 (reader 1) and 28 (reader 2) benign lesions were classified as malignancies on rs-EPI DWI, including: intraductal papilloma (*n* = 10 and 8 for reader 1 and reader 2, respectively), fibroadenoma/fibroadenomatous hyperplasia (*n* = 5 and 6), inflammatory change (*n* = 5 and 7) (Figures 5e–h), adenosis (*n* = 7 and 6), fibromatosis (*n* = 1 and 1), and phyllodes tumor (*n* = 1 and 0).

## Discussion

Readout-segmented echo-planar imaging DWI shows potential in breast cancer screening and diagnosis. In our study, rs-EPI DWI can detect about 90% of breast lesions identified by DCE-MRI. The sensitivity, specificity, and negative predictive value (NPV) of rs-EPI DWI for distinguishing breast lesions are comparable to those of DCE-MRI.

Non-contrast DWI has shown the potential to detect and differentiate breast lesions without the long-term toxicities potentially associated with contrast dosing. However, reported sensitivities (from 45 to 94%) and specificities (from 79 to 95.7%) varied greatly in earlier studies (34–37). Recently, several studies demonstrated improved diagnostic performance when using the readout-segmented technique (10, 19, 38).

In this study, we intended to explore the feasibility of rs-EPI DWI as an imaging tool for breast cancer screening, in particular in women with high breast density. For this purpose, rs-EPI DWI should firstly achieve the ability to detect lesions as many as possible, in particular for non-cystic lesions, which are of higher risk of malignancy. DCE-MRI is the most sensitive imaging modality for breast cancer detection and has an excellent spatial resolution. Based on our results, rs-EPI DWI can detect about 90% of the breast lesions identified by DCE-MRI, even with a slice thickness of 5.0 mm. The detection ability was slightly lower than the result reported by Telegrafo et al. (37) using an unenhanced-MRI protocol of short TI inversion recovery (STIR), T2-weighted and DWI (90% vs. 96%), which may be due to the thicker slice thickness of DWI in our study (5.0 mm vs. 3.0 mm). Small and benign lesions on DCE-MRI may be more easily overlooked by rs-EPI DWI. Of those missed lesions, however, most were rated as BI-RADS 2 or 3 on DCE-MRI, and only two of these lesions were finally verified as malignancies based upon histopathological examination. Therefore, although rs-EPI DWI may overlook some breast lesions identified by DCE-MRI, the probability of missing breast malignancies was quite low.

The second ability that rs-EPI DWI should reach is to pick up suspiciously malignant lesions. Thus, we included pathological results as a reference to evaluate the performance of DCE-MRI and rs-EPI DWI in distinguishing breast cancers from benign diseases. During identifying breast cancers, rs-EPI DWI not only provided quantitative parameters (ADC values) but also detailed visualization of lesion morphological characteristics. By integrating each of these parameters together into a comprehensive diagnostic protocol, the performance of rs-EPI DWI for distinguishing breast lesions was actually equivalent to that of DCE-MRI, which was also provided by previous studies (10, 37). The sensitivity of DWI in our study was a little higher than that reported by Bickelhaupt et al. (17) (95.9%−97.7% vs. 92.0%), which may be partly due to the larger mean lesion size of our study. There were different causes for the inclusion into our study, such as clinical symptoms, which may explain the larger lesion size compared to the study using only patients with suspicious x-ray mammography (17).

Although encouraging results were found, several malignancies were still diagnosed as benign diseases according to rs-EPI DWI alone. Some small breast cancers (<10 mm) showed a relatively well-defined margin and homogeneous internal structures, and ROI of those lesions for quantitative measurements may be inaccurate due to partial volume effects. These factors may have led to the false classification of some small malignant lesions by rs-EPI-DWI. Some difficulties were also found when attempting to distinguish between the breast fibrocystic hyperplasia and breast cancers. In this study, a pathologically proven breast mucinous carcinoma was characterized as fibrocystic hyperplasia in a 46-year-old woman by an experienced radiologist (Figures 5a–d). This lesion had an irregular shape and heterogeneously increased T2 signal intensity with a high ADC value, thus resembling a manifestation of the breast fibrocystic hyperplasia. Conversely, some cases of the benign disease were wrongly interpreted as malignancies by the readers when only rs-EPI DWI data were used for the diagnosis. For example, a granulomatous mastitis, presenting as a large lesion with an irregular shape, heterogeneous internal structures, and decreased ADC value, was misdiagnosed as breast cancer (Figures 5e–h). It was also difficult to accurately identify non-mass-like lesions due to irregular distribution and inaccurate measurements of the ADC value. In this context, clinical symptoms and signs, and enhancement characteristics on DCE-MRI may provide additional information for the differential diagnosis.

Several limitations existed in our study. First, this study was conducted retrospectively at a single center. Second, the spatial resolution for the breast DWI in our study (5.0 mm) was lower than that of DCE-MRI (1.5 mm), which may result in missing some small lesions. In order to act as a reliable screening tool, the spatial resolution of the breast DWI needs to be further improved. Newly explored simultaneous multi-slice (SMS) acquisition based on the blipped controlled aliasing in parallel imaging results in the higher acceleration (blipped CAIPIRINHA) technique (39). The latter method has the potential to substantially reduce acquisition time and make it possible to improve the spatial resolution (smaller than 5.0 mm), without requiring additional scan time. Lastly, MR examinations in this study were performed using two types of the breast coils because of a system update and different scanning protocols were used for DCE-MRI, which may have introduced some variations in the results. Thus, a future multi-center clinical study using optimized standard MR sequences should be performed to further validate these results for rs-EPI DWI in the breast cancer screening and diagnosis.

In conclusion, rs-EPI DWI can detect about 90% of breast lesions identified with DCE-MRI, and provides comparable diagnostic performance to that of DCE-MRI for characterizing breast cancers. These findings suggest that rs-EPI DWI may provide a safe and reliable supplemental imaging modality for breast cancer screening, particularly for patients with dense breasts and contraindication for GBCA.

## Data Availability Statement

The raw data supporting the conclusions of this article will be made available by the authors, without undue reservation.

## Ethics Statement

The studies involving human participants were reviewed and approved by The institutional review board of Tongji Hospital, Tongji Medical College, Huazhong University of Science and Technology. Written informed consent for participation was not provided by the participants' legal guardians/next of kin because: This is a retrospective study and no extra imaging scans and intervention were performed for participants. A waived written informed consent was approved by Our institutional review board.

## Author Contributions

ZLY and YQH performed study design, information collection, statistical analysis, and manuscript editing. LMX and TA guided study design, reviewed images, and revised manuscript. MXZ, XYZ, and HTZ provided technical support and software for measuring ADC values. JH and CAZ collected images and clinical information. All authors contributed to the article and approved the submitted version.

## Conflict of Interest

XYZ and HTZ were employed by the company Siemens Healthcare. The remaining authors declare that the research was conducted in the absence of any commercial or financial relationships that could be construed as a potential conflict of interest.

## Abbreviations

DWI: diffusion-weighted imaging
rs-EPI: readout-segmented echo-planar imaging
DCE-MRI: dynamic contrast-enhanced MRI
BI-RADS: Breast Imaging Reporting and Data System
ADC: apparent diffusion coefficient
PPV: positive predictive value
NPV: negative predictive value
GBCAs: gadolinium-based contrast agents
FGT: fibroglandular tissue
BPE: background parenchymal enhancement
TIC: time-signal intensity curve
ER: estrogen receptor
PR: progesterone receptor
HER2: human epidermal growth factor receptor-2
ROI: region of interest.
