# Supplementary material for: Detection and Classification of Breast Lesions With Readout-Segmented Diffusion-Weighted Imaging in a Large Chinese Cohort
Source: Front Oncol. 2021 Mar 22;11:636471. doi: 10.3389/fonc.2021.636471 (PMC8020903; doi:10.3389/fonc.2021.636471)
Supplement: Supplementary file 1 [file Table_1.docx]

**Supplementary Materials**

**Supplementary Table 1: Diagnostic criteria for rs-EPI DWI and DCE-MRI in characterization of breast lesions.**

| **Modality** | **Characteristic** | **Benign lesion** | **Malignant lesion** |
| --- | --- | --- | --- |
| **rs-EPI DWI** | ADC | >1.25 x 10^−3^ mm^2^/sec | ≤1.25 x 10^−3^ mm^2^/sec |
|  | Margin | Well-defined | Ill-defined |
|  | Internal signal | Homogeneous | Heterogeneous |
| **DCE-MRI** | Time-signal intensity curve | Persistent | Washout, plateau |
|  | Morphology (mass) | Regular (oval, round) | Irregular |
|  | Margin (mass) | Well-defined | Ill-defined |
|  | Internal enhancement (mass) | Homogeneous, septation | Heterogeneous |
|  | Distribution (non-mass) | Diffuse, regional, focal | Segmental, linear |
|  | Internal enhancement (non-mass) | Homogeneous | Heterogeneous |

DCE-MRI= dynamic contrast-enhanced magnetic resonance imaging, rs-EPI DWI= readout-segmented echo-planar imaging diﬀusion-weighted imaging, ADC=apparent diffusion coefficient. Reference 10 (10).

**Supplementary Table 2: Breast lesions’ characteristics on DCE-MRI and the corresponding diffusion parameters.**

|  | **Benign lesions (n=203)** | **Malignant lesions (n=220)** | **P value** |
| --- | --- | --- | --- |
| **Mass-like lesions** | n=192 (186 enhanced lesions and 6 non-enhanced lesions)* | n=181 |  |
| Size (mm) | 15.1±17.7 (10)  [12.5, 17.7] | 23.5±15.0 (18)  [21.3, 25.7] | <0.001 |
| Shape |  |  | <0.001 |
| Round, oval | 60.2% (112/186) | 6.6% (12/181) |  |
| Irregular | 39.8% (74/186) | 93.4% (169/181) |  |
| Margin |  |  | <0.001 |
| Well-defined | 91.4% (170/186) | 27.1% (49/181) |  |
| Ill-defined | 8.6% (16/186) | 72.9% (132/181) |  |
| Internal enhancement |  |  | <0.001 |
| Homogeneous | 60.8% (113/186) | 15.5% (28/181) |  |
| Heterogeneous | 39.2% (73/186) | 84.5% (153/181) |  |
| **Non-mass-like lesions** | n=11 | n=39 |  |
| Distribution |  |  | 0.205 |
| Diffuse, regional, focal | 54.5% (6/11) | 28.2% (11/39) |  |
| Segmental, linear | 45.5% (5/11) | 71.8% (28/39) |  |
| **Time-signal intensity curve** |  |  | <0.001 |
| Persistent | 52.2% (106/203) | 2.7% (6/220) |  |
| Plateau | 31.0% (63/203) | 45.9% (101/220) |  |
| Washout | 7.9% (16/203) | 50.9% (112/220) |  |
| Slight or no enhancement | 8.9% (18/203) | 0.5% (1/220) |  |
| **Diffusion parameters** |  |  |  |
| Mean ADC | 1.21±0.37 | 0.85±0.17 | <0.001 |

DCE-MRI= dynamic contrast-enhanced magnetic resonance imaging, ADC=apparent diffusion coefficient.

*The 6 lesions were non-enhanced on DCE-MRI, while they were visible on rs-EPI-DWI and manifested as mass-like lesions.

**Supplementary Table 3: Performance of rs-EPI DWI and DCE-MRI in diagnosing breast cancer on a per-patient basis with pathology as reference standard.**

|  | **Results (n)** | | | |  | **Test performance (%)** | | | | |
| --- | --- | --- | --- | --- | --- | --- | --- | --- | --- | --- |
| **Imaging modality** | **TP** | **TN** | **FP** | **FN** |  | **Sens.** | **Spec.** | **PPV** | **NPV** | **Acc.** |
| **rs-EPI DWI** |  |  |  |  |  |  |  |  |  |  |
| **Overall** |  |  |  |  |  |  |  |  |  |  |
| R1 | 199 | 97 | 23 | 8 |  | 96.1 (199/207)  [92.5-98.0] | 80.8 (97/120)  [72.9-86.9] | 89.6 (199/222)  [84.9-93.0] | 92.4 (97/105)  [85.7-96.1] | 90.5 (296/327)  [86.9-93.2] |
| R2 | 202 | 97 | 23 | 5 |  | 97.6 (202/207)  [94.5-99.0] | 80.8 (97/120)  [72.9-86.9] | 89.8 (202/225)  [85.1-93.1] | 95.1 (97/102)  [89.0-97.9] | 91.4 (299/327)  [87.9-94.0] |
| **Amount of FGT*** | | |  |  |  |  |  |  |  |  |
| **a/b** | | |  |  |  |  |  |  |  |  |
| R1 | 56 | 19 | 4 | 1 |  | 98.2 (56/57)  [90.7-99.7] | 82.6 (19/23)  [62.9-93.0] | 93.3 (56/60)  [84.1-97.4] | 95.0 (19/20)  [76.4-99.1] | 93.8 (75/80)  [86.2-97.3] |
| R2 | 57 | 17 | 6 | 0 |  | 100 (57/57)  [93.7-100] | 73.9 (17/23)  [53.5-87.5] | 90.5 (57/63)  [80.7-95.6] | 100 (17/17)  [81.6-100] | 92.5 (74/80)  [84.6-96.5] |
| **c/d** | | |  |  |  |  |  |  |  |  |
| R1 | 143 | 78 | 19 | 7 |  | 95.3 (143/150)  [90.7-97.7] | 80.4 (78/97)  [71.4-87.1] | 88.3 (143/162)  [82.4-92.4] | 91.8 (78/85)  [84.0-96.0] | 89.5 (221/247)  [85.0-92.7] |
| R2 | 145 | 80 | 17 | 5 |  | 96.7 (145/150)  [92.4-98.6] | 82.5 (80/97)  [73.7-88.8] | 89.5 (145/162)  [83.8-93.3] | 94.1 (80/85)  [87.0-97.5] | 91.1 (225/247)  [86.9-94.0] |
| **DCE-MRI** |  |  |  |  |  |  |  |  |  |  |
| **Overall** |  |  |  |  |  |  |  |  |  |  |
| R1 | 203 | 94 | 26 | 4 |  | 98.1 (203/207)  [95.1-99.3] | 78.3 (94/120)  [70.2-84.8] | 88.6 (203/229)  [83.9-92.1] | 95.9 (94/98)  [90.0-98.4] | 90.8 (297/327)  [87.2-93.5] |
| R2 | 205 | 85 | 35 | 2 |  | 99.0 (205/207)  [96.6-99.7] | 70.8 (85/120)  [62.2-78.2] | 85.4 (205/240)  [80.4-89.3] | 97.7 (85/87)  [92.0-99.4] | 88.7 (290/327)  [84.8-91.7] |
| **Amount of FGT*** |  |  |  |  |  |  |  |  |  |  |
| **a/b** |  |  |  |  |  |  |  |  |  |  |
| R1 | 57 | 19 | 4 | 0 |  | 100 (57/57)  [93.7-100] | 82.6 (19/23)  [62.9-93.0] | 93.4 (57/61)  [84.3-97.4] | 100 (19/19)  [83.2-100] | 95.0 (76/80)  [87.8-98.0] |
| R2 | 57 | 17 | 6 | 0 |  | 100 (57/57)  [93.7-100] | 73.9 (17/23)  [53.5-87.5] | 90.5 (57/63)  [80.7-95.6] | 100 (17/17)  [81.6-100] | 92.5 (74/80)  [84.6-96.5] |
| **c/d** |  |  |  |  |  |  |  |  |  |  |
| R1 | 146 | 75 | 22 | 4 |  | 97.3 (146/150)  [93.3-99.0] | 77.3 (75/97)  [68.0-84.5] | 86.9 (146/168)  [81.0-91.2] | 94.9 (75/79)  [87.7-98.0] | 89.5 (221/247)  [85.0-92.7] |
| R2 | 148 | 68 | 29 | 2 |  | 98.7 (148/150)  [95.3-99.6] | 70.1 (68/97)  [60.4-78.3] | 83.6 (148/177)  [77.5-88.3] | 97.1 (68/70)  [90.2-99.2] | 87.4 (216/247)  [82.7-91.0] |

TP= true positive, TN=true negative, FP=false positive, FN=false negative, Sens.=sensitivity, Spec.=specificity, PPV= positive predictive value, NPV=negative predictive value, Acc.= Accuracy, R1=reader 1, R2=reader 2, rs-EPI DWI=readout-segmented echo-planar imaging diﬀusion-weighted imaging, DCE-MRI=dynamic contrast-enhanced magnetic resonance imaging, FGT= fibrograndular tissue.

Data in parentheses are the numerator and denominator. Data in brackets are 95% confidence intervals.

*The amount of FGT included almost entirely fat (a), scattered fibroglandular tissue (b), heterogeneous fibroglandular tissue (c), extreme fibroglandular tissue (d).

**Supplementary Table 4: Mean ADC values of invasive breast cancers with different molecular subtypes.**

|  | **Mean ADC**  (×10^-3^mm^2^/s) | **P values** |
| --- | --- | --- |
| **Molecular subtypes** |  | 0.032 |
| luminal A tumor (n=21) | 0.81±0.11 |  |
| luminal B tumor (n=71) | 0.81±0.13 |  |
| HER2-enriched tumor (n=56) | 0.86±0.14 |  |
| Triple-negative tumor (n=28) | 0.88±0.13 |  |
| **Subgroup comparison** |  | 0.072 |
| Triple-negative tumor (n=28) | 0.88±0.13 |  |
| Non-triple-negative tumor (n=148) | 0.83±0.14 |  |
| **Subgroup comparison** |  | 0.003 |
| Luminal tumor (n=92) | 0.81±0.13 |  |
| Non-luminal tumor (n=84) | 0.87±0.14 |  |

HER2=Human epidermal growth factor receptor-2.
